# Ustekinumab in pediatric patients with Crohn’s disease: safety, and efficacy results from a multicenter retrospective study in China

**DOI:** 10.3389/fped.2024.1371322

**Published:** 2024-04-11

**Authors:** Ping Li, Lin Wang, Zifei Tang, Yuhuan Wang, Zhanju Liu, Wensong Ge, Ying Huang

**Affiliations:** ^1^ Department of Gastroenterology, National Children’s Medical Center, Children’s Hospital of Fudan University, Shanghai, China; ^2^ Department of Gastroenterology, The Shanghai Tenth People’s Hospital, Tongji University, Shanghai, China; ^3^ Department of Gastroenterology, Xinhua Hospital, School of Medicine, Shanghai Jiao Tong University, Shanghai, China

**Keywords:** Crohn's disease, ustekinumab, pediatric, efficacy, safety

## Abstract

**Background:**

Ustekinumab (UST) is approved as an effective therapy for Crohn's disease (CD) in adults. Off-label use is increasing in the pediatric population, more data on safety and efficacy in pediatric patients with CD is urgently needed.

**Aims:**

This study aimed to evaluate the clinical efficacy and safety of UST in children and adolescents with Crohn's disease.

**Methods:**

This multicenter retrospective study carried out at three tertiary care centers, and identified children who received their first dose of UST at 18 years old or younger and followed up for a minimum of 24 weeks. Data on demographics, disease behavior, location and activity, treatment history were collected. The primary outcomes were clinical remission at weeks 24–32 and weeks 48–56 of UST therapy. Secondary outcomes were clinical response at the same time points, endoscopic remission, changes in C-reactive protein (CRP), erythrocyte sedimentation rate (ESR), albumin and fecal calprotectin, improvement in growth parameters, and rate of adverse events.

**Results:**

Sixteen patients were included, and 11/13 (84.6%) continued to receive UST after 1 year. Our data demonstrate that the clinical remission rates were 41.7% at weeks 24∼32 with the Weighted pediatric CD activity index (wPCDAI) was lower than baseline (43.8, IQR: 31.3–51.9 vs.15, IQR: 5.6–25, *p* < 0.001) and 75% at weeks 48–56 with wPCDAI was lower than baseline (42.5, IQR: 23.8–50 vs. 7.5, IQR: 0–13.8, *p* = 0.004). Five of eleven children achieved endoscopic remission. No serious adverse events were recorded during the study period.

**Conclusions:**

UST is efficacious and safe in pediatric patients with CD. Pediatric patients could benefit from UST as either a primary or secondary biologic therapy for the induction, or maintenance of remission of CD.

## Introduction

Crohn's disease (CD) is a chronic inflammatory disease with unknown etiology that destroys the gastrointestinal tract (1). Patients often have multiple complications, which seriously affect the quality of life. Compared with adults, pediatric patients with CD have fewer drug choices, more difficult treatment, higher cost, poor prognosis, and need more therapeutic drugs to choose. Despite the increasing number of available treatments, anti-tumor necrosis factor (TNF) agents remain the only biological therapy for pediatric patients with moderate to severe CD (2, 3). Despite their recognized effectiveness, the primary non-response rate is 10%–30% and the secondary loss of response rate is 13%–40% (4–7).

In recent years, new biologics have been approved for the treatment of CD in adults. Foreign studies have shown that ustekinumab (UST) has high clinical remission rate and safety on CD in the treatment of tumor necrosis factor (TNF-α) antibody loss in adults, but there is little experience in treating CD in children (8, 9). At present, the mechanism of action of UST in the treatment of CD is not very clear. The main mechanism may be that UST is a fully humanized IgG1 monoclonal antibody that binds with specificity to the p40 protein subunit of interleukins IL-12 and IL-23 blocks the inflammatory pathway mediated by downstream Th1 cells and Th17 cells (10, 11). As we have seen previously with biologics first marketed in the adult Inflammatory bowel disease (IBD) population, there is increasing off-label use of UST in children even in the absence of pediatric pharmacokinetic data. Data on the effectiveness of UST in the pediatric population is limited. However, with increasing off-label use in children, more data on safety and effectiveness in children with IBD are needed. Herein we report our experience on the clinical effectiveness and safety of UST in the treatment of CD in children and adolescents from three tertiary care IBD referral center in China.

## Methods

### Patients

This study was a retrospective multicenter study carried out at three tertiary care centers in Shanghai, China. We reviewed a database of pediatric IBD patients diagnosed before age 18. As of November 2023, patients with CD who received their first dose of UST at 18 years old or younger and followed up for a minimum of 24 weeks were included in this study. All patients were cared for by a board-certified gastroenterologist, and UST was prescribed at the discretion of the prescribing physician. Ethics approval for the study was obtained from the ethics committee of each center. Informed consent for participation and sample collection was obtained from their parents.

### Data collection

Electronic medical records were reviewed for demographic, medical and surgical treatment history, including steroid use, past biologics and immunomodulator exposure, disease location and behavior (Paris classification) (12), and disease activity (Weighted pediatric CD activity index [wPCDAI] (13). Clinical activity scores and associated laboratory data were recorded at baseline (ustekinumab initiation time) and at 24–32 and 48–56 weeks of follow-up. Laboratory data included complete blood count, albumin, erythrocyte sedimentation rate (ESR), C-reactive protein (CRP), and fecal calprotectin. Considering the different assays used to assess CRP, we believe that this serum marker is elevated when the value ≥8 mg/L. We also collected data on adverse events, including reactions at infusion and injection sites, and serious adverse events (SAEs).

### Disease activity assessments and outcome measures

Clinical evaluation was performed before induction treatment and at weeks 24–32, and 48–56 of UST therapy via wPCDAI with values indicating clinical remission (<12.5 points). The clinical response, defined as a decrease in wPCDAI >17.5 (13, 14). Colonoscopy was performed at week 0 and weeks 24–32 or weeks 48–56 of UST therapy to assess mucosal healing by using a simple endoscopic CD score (SES-CD). Endoscopic remission was defined as SES-CD <3 points and a decline in the SES-CD of >50% was defined as endoscopic response (15). In addition, we assessed changes in height, weight, and body mass index (BMI) between baseline to 12 months using a Z-score based on WHO standards.

The primary outcomes were clinical remission at weeks 24–32 and weeks 48–56 of UST therapy. Secondary outcomes were clinical response at the same time points, endoscopic remission, changes in CRP, ESR, albumin and fecal calprotectin, improvement in growth parameters.

### Date analyses

Statistical analysis was performed using SPSS 20.0 for Windows (IBM, Somers, NY). Continuous clinical and demographic variables are expressed as the median and interquartile range (IQR). The categorical variables are expressed as percentages. The Mann–Whitney test was applied for the comparison of two groups. *P* value <0.05 was considered statistically significant.

## Results

### Patient characteristics and UST regimens

From the three hospitals, we identified 16 patients with CD who received UST treatment. The demographic information and disease characteristics of those patients are summarized in Table 1. Among three Ala, two were very early-onset IBD (VEO-IBD), and one of the cases with IL-10 gene defect confirmed by whole exome sequence. Among the 13 cases of Alb, one with the defect of BTK gene confirmed by whole exome sequence. Except for 5 bio-naïve patients whose parents actively chose UST, all others (68.8%) received alternative biologics before UST treatment. Only one patient was treated with corticosteroids accompany, and the corticosteroids were discontinued within 16 weeks of UST treatment. At baseline, while 12 patients were in the active phase of the disease and the remaining four were in clinical remission following receiving exclusive enteral nutrition (EEN) therapy. However, one patient still had intestinal stenosis and capsule endoscopy incarceration after EEN treatment, other three patients had active disease on colonoscopy, which warranted the initiation of UST. Two patients discontinued UST at week 32 due to the poor response. Among the 13 cases who had been followed more than 1 year, 11/13 (84.6%) remained on UST treatment.

**Table 1:** Demographics and clinical characteristics of pediatric crohn’s disease patients at initiation of UST.

Patient characteristics	*n* = 16
Male, *n* (%)	11 (68.8)
Median age (IQR) at diagnosis (year)	12.8 (10.2–13.5)
Median age (IQR) at initiation of UST (year)	14.0 (11.2–15.5)
Median (IQR) from diagnosis to initiation of UST (month)	15.9 (8.5–28.0)
Median (IQR) observed duration UST (week)	56.5 (34.5–61.8)
Age at diagnosis (Paris classification) (%)	
A1a	3 (18.8)
A1b	13 (81.3)
Disease location (Paris classification), *n* (%)	
L1	1 (6.3)
L2	2 (12.5)
L3	11 (68.8)
L4a	4 (25.0)
L4b	9 (56.3)
Perianal	5 (31.3)
Disease phenotype (Paris classification)	
B1	6 (37.5)
B2	8 (50.0)
B3	1 (6.3)
Growth failure, *n* (%)	2 (12.5)
Extraintestinal manifestations, *n* (%)	3 (18.8)
Previous surgery, *n* (%)	2 (12.5)
Previous biological therapy, *n* (%)	
Infliximab	11 (68.8)
Adalimumab	2 (12.5)
Vedolizumab	1 (6.3)
Total number of biologicals previously exposed, *n* (%)	
None	5 (31.3)
One	8 (50.0)
Two	3 (18.8)
Other therapy before UST, *n* (%)	
EEN	14 (87.5)
5-ASA	11 (68.8)
Corticosteroids	6 (37.5)
Azathioprine	3 (18.8)
Methotrexate	4 (25.0)
Thalidomide	2 (12.5)
Intravenous gamma globulin	1 (6.3)
Indication of UST, *n* (%)	
Nonresponse to biologicals	11 (68.8)
Patient chose UST	5 (31.3)
wPCDAI (median, IQR)	42.5 (10.0–50.0)
SES-CD (median, IQR)	10 (4.0–16.8)
CRP, mg/L (median, IQR)	8.3 (1.8–37.6)

IQR, interquartile range; UST, ustekinumab; A1a, IBD diagnosed before age 10 years; A1b, IBD diagnosed at age ≥10 years but before age17 years; L1, involving one third of the distal ileum only with limited or no cecal disease; L2, colonic involvement only; L3, involvement of both the terminal ileum and colon; L4a, esophagogastroduodenal disease; L4b, involvement of the jejunum and/or proximal two-third of the ileum; B1, non-stricturing, non-penetrating disease; B2, stricturing disease; B3,penetrating disease excluding isolated perianal or rectovaginal fistulas; 5-ASA, 5-aminosalicylic acid; EEN, exclusive enteral nutrition; CRP, C-reactive protein; wPCDAI, weighted pediatric Crohn's disease activity index; SES-CD, simple endoscopic score for Crohn's disease.

Because UST has not been approved for children with CD, the dose and interval between doses refers to the regimen approved in Chinese adult CD patients (induction with 260 mg for patients <55 kg, 390 mg for patients ≤85 kg, and 520 mg for patients >85 kg, intravenously, maintenance with 90 mg every 8–12 weeks, subcutaneously) and Phase 1 clinical trial of UST in children with moderate to severe active CD (16). Patients older than 15 years of age or weighing more than 40 kg received an adult dose of UST. While the others received a first induction dose of 6–9 mg/kg intravenously at week 0 and followed by an intravenous infusion of 6–9 mg/kg for maintenance every 4–8 weeks, or a subcutaneous infusion of 90 mg/dose for maintenance every 8–12 weeks. In summary, Table 2 summarizes UST treatment information including dose and administration method at induction, the mode, frequency, and duration of maintenance therapy administration. Referring to the relevant literature in adults and children, all patients received a first induction dose of 6–9 mg/kg intravenously. And then according to the disease activity and the wishes of their families, five patients followed by a subcutaneous infusion for maintenance every 8–12 weeks. Other eleven patients received an intravenous infusion for maintenance, including 10 of them at an interval of 8 weeks, and one patient received continuous intravenous infusion maintenance every 4 weeks due to severe disease activity and multi-segmental stenosis of the small intestine. During the follow-up period, one patient needed to shorten the interval to every 6 weeks. One child was still in clinical activity after shortening to 4 weeks, so treatment with UST was discontinued at 32 weeks.

**Table 2:** UST therapy of the study cohort (*n* = 16).

UST induction dose, mg/kg (median, IQR)	7.0 (5.7–7.5)
UST maintenance therapy	
IV maintenance, *n* (%)	11 (68.8)
Dose, mg/kg (median, IQR)	7.5 (6.0–8.9)
SC maintenance, *n* (%)	5 (31.3)
Dose, mg	90
Frequency of maintenance therapy, *n* (%)	
Every 4 week	1 (6.3)
Every 8 week	13 (81.3)
Every 12 week	2 (12.5)
Concomitant therapy, *n* (%)	
5-ASA	8 (50.0)
Corticosteroids	1 (6.3)
Azathioprine	2 (12.5)
Methotrexate	4 (25.0)
Thalidomide	2 (12.5)
Vedolizumab	1 (6.3)
Intravenous gamma globulin	1 (6.3)

IQR, interquartile range; 5-ASA, 5-aminosalicylic acid; EEN, exclusive enteral nutrition; UST, ustekinumab; IV, intravenous; SC, subcutaneous.

### Clinical outcomes

**Figure 1:**
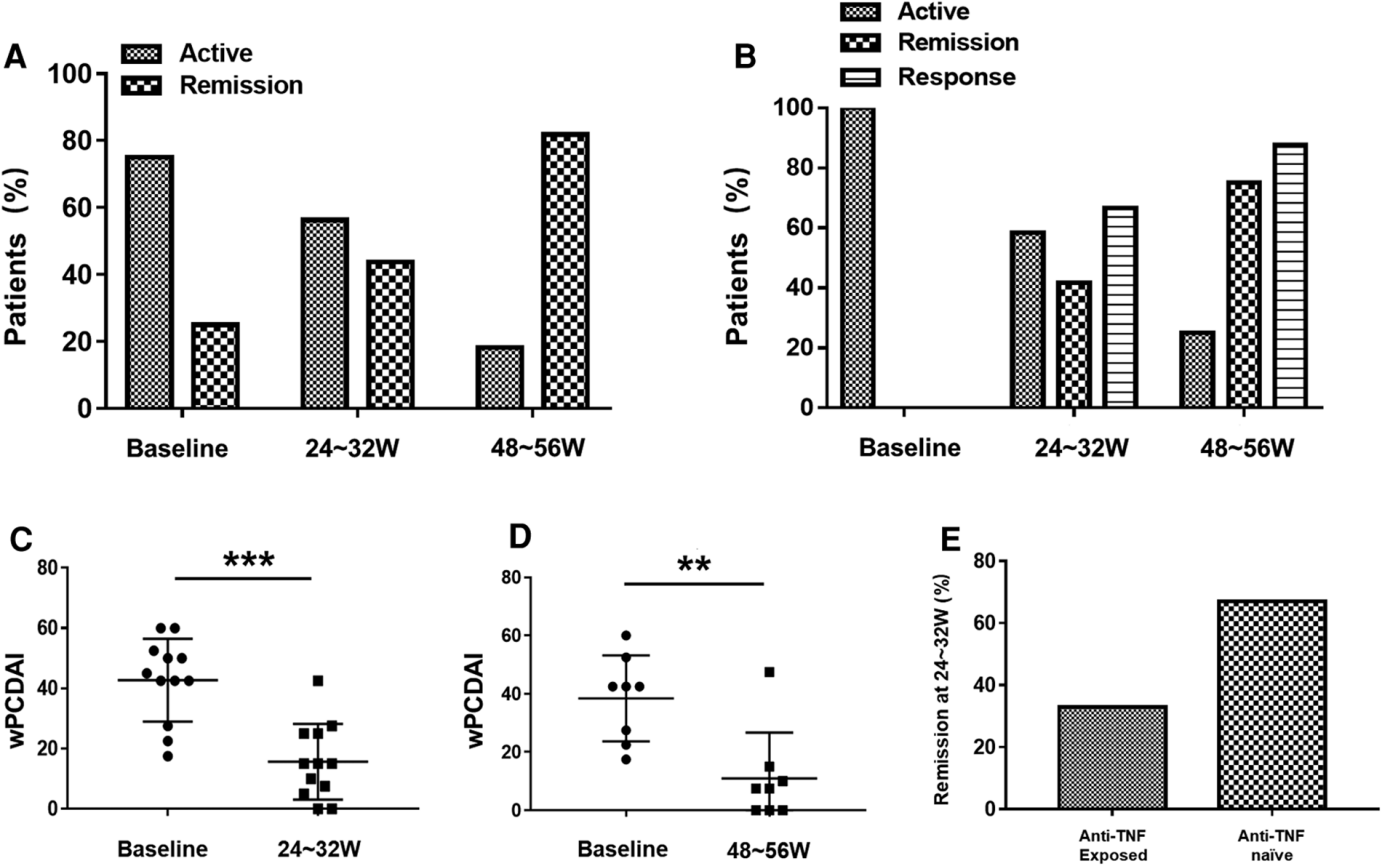
(**A**) Proportion of patients in clinical remission and active phase under ustekinumab treatment (at baseline, weeks 24–32 and weeks 48–56). (**B**) Proportion of patients (wPCDAI > 12.5 at baseline) in clinical remission, clinical response and active phase under ustekinumab treatment (at baseline, weeks 24–32 and weeks 48–56). Changes in wPCDAI at baseline and weeks 24–32 (**C**) or baseline and weeks 48–56 (**D**) of patients who were clinical active at baseline. (**E**) Ustekinumab remission rates at weeks 24–32 in anti-TNF exposed vs. biologic-naïve Crohn disease patients. wPCDAI = weighted pediatric Crohn's disease activity index, TNF = tumor necrosis factor. **P < 0.01, ***P < 0.001.

Four patients (25%) had a wPCDAI (<12.5) indicating clinical remission at baseline. However, one patient still had intestinal stenosis and capsule endoscopy incarceration after EEN treatment, other three patients had active disease on colonoscopy, which warranted the initiation of UST. As shown in Figure 1A, the clinical remission improved to 56.3% at 24–32 weeks and to 81.8% at 48–56 weeks of UST treatment. In addition, among patients who were clinical active at baseline, there was a significant decrease in wPCDAI at 24–32 weeks of UST treatment (43.8, IQR: 31.3–51.9 vs.15, IQR: 5.6–25, *p* < 0.001) (Figure 1C). At 24–32 weeks of UST therapy, 8/12 patients (66.7%) achieved clinical response, and 5/12 patients (41.7%) achieved clinical remission. Among patients followed over 48–52 weeks, 7/8 patients (87.5%) achieved clinical response, and 6/8 patients (75%) achieved clinical remission at 48–56 weeks (Figure 1B) and wPCDAI was significantly lower than baseline (42.5, IQR: 23.8–50 vs. 7.5, IQR: 0–13.8, *p* = 0.004) (Figure 1D).

Eleven patients had been exposed to anti-TNF therapy before starting UST, of which two patients had been on ≥2 anti-TNF. The cause for discontinuation of anti-TNF included secondary loss of response (*n* = 10) and primary nonresponse to anti-TNF (*n* = 1). Bio-naïve patients were more likely to achieve clinical remission than bio-exposed patients 66.7% vs. 33.3% at 24–32 weeks, respectively (Figure 1E).

### Endoscopic outcomes

**Figure 2:**
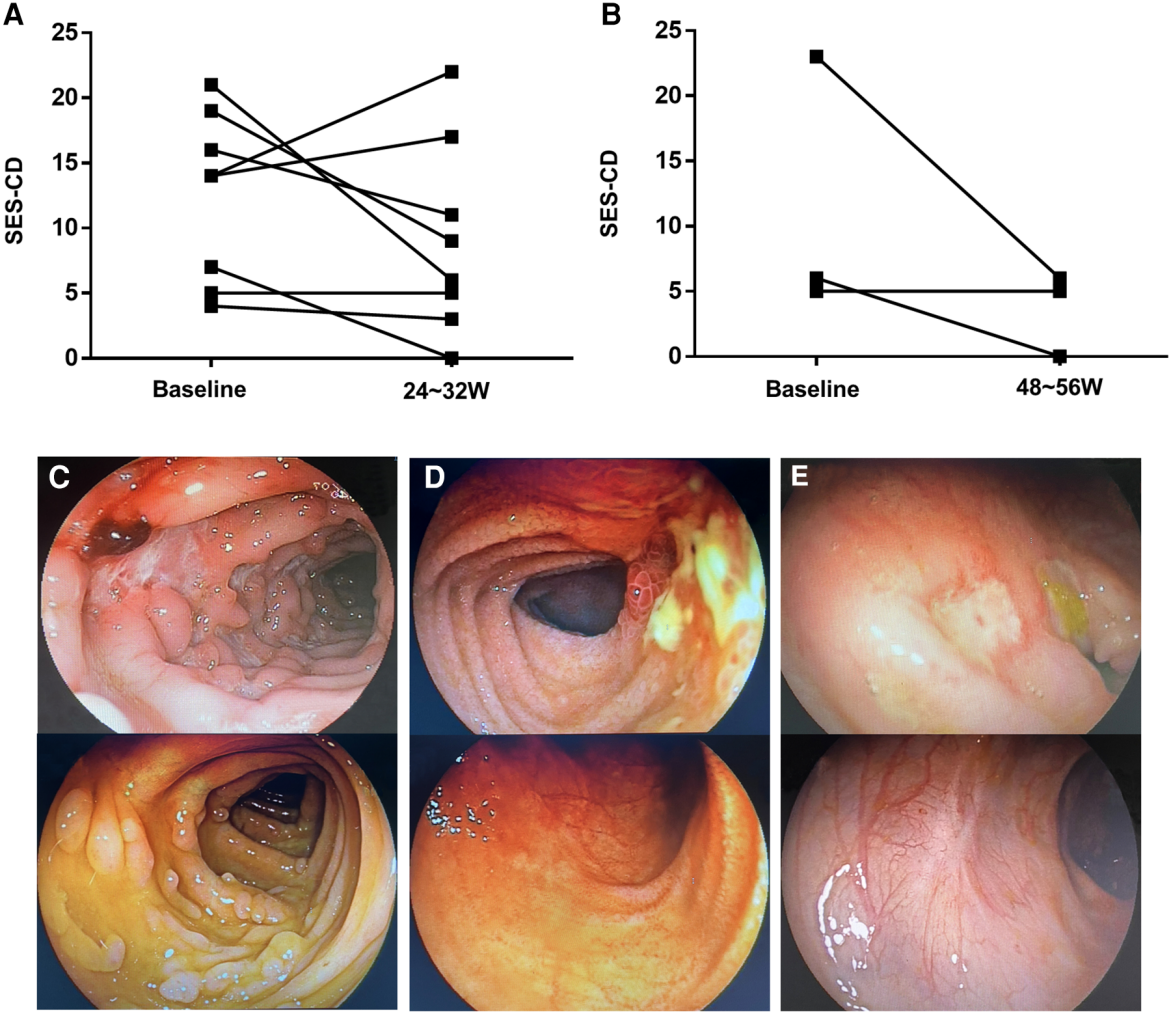
(**A**) Changes in the simple endoscopic score (SES-CD) of eight patients at weeks 24–32. (**B**) Changes in the SES-CD of three patients at weeks 48–56. (**C**) Transverse colon findings in a 11-year-old girl (at week 0, at week 24). (**D**) Findings of terminal ileum in a 12-year-old girl (at week 0 and at week 26). (**E**) Findings of descending colon in a 3-year-old boy (at week 0 and at week 27).

All patients underwent colonoscopy at baseline. During follow-up, colonoscopy were performed with scoring of SES-CD in 11 patients. Of those, eight were performed at 24–32 weeks and three at 48–56 weeks. Figures 2A,B shows the change in SES-CD. Among the eight patients evaluated at week 24–32, 1/8 patients (12.5%) achieved endoscopic remission and 3/8 patients (37.5%) achieved endoscopic response. Three patients were evaluated at weeks 48–56, of whom one (33.3%) achieved endoscopic remission and two (66.7%) achieved endoscopic response. Figures 2C–E shows the change in endoscopy findings of three patients who achieved clinical response during follow-up.

### Biologic outcomes

At baseline, eight (50%) patients had a normal CRP. Among the eight with an elevated baseline CRP, the test normalised in four at 24–32 weeks. 9/11 (81.8%) patients who were followed over 48–56 weeks achieved normal CRP at 48–56 weeks. The longitudinal changes of biological indicators at baseline and during maintenance therapy are shown in Table 3. It can be seen that ESR and fecal calprotectin decreased significantly at 24–32 weeks and 48–56 weeks after UST treatment. Serum albumin levels and platelet counts were significantly improved at 48–56 weeks compared to baseline. White blood cell count, hemoglobin and CRP levels did not change significantly over time.

**Table 3:** Summary of clinical indicators at baseline, at 24∼32 weeks and at 48∼56 weeks.

	*n* = 16			*n* = 11		
	Baseline	24–32 weeks	*P* value	Baseline	48–56 weeks	*P* value
CRP, mg/L (median, IQR)	8.3 (1.8–37.6)	3.0 (0.5–8.9)	0.21	6.6 (1.6–32.1)	2.3 (0.5–8)	0.16
ESR, mm/h (median, IQR)	43 (27.5–84.8)	20 (12–54.5)	0.02	43 (27–86)	19 (5–32)	0.003
FC, ug/g (median, IQR)	346.5 (239.4–757.9)	230 (49.8–365.4)	0.02	342 (231.7–778.5)	64.9 (40.1–173)	0.01
Albumin, g/L (median, IQR)	38.9 (36.2–42.2)	40.9 (38–42.5)	0.27	39.2 (36.4–42.1)	44.3 (41.8–45.1)	0.009
Hemoglobin, g/L (median, IQR)	119 (104–141.8)	130.5 (109.8–140.5)	0.86	130 (110–142)	129 (124–145)	0.23
WBC,10^9 ^/L (median, IQR)	6.6 (4.7–10.2)	7.2 (5.2–8.7)	0.75	6.6 (4.7–8.1)	6.3 (5.0–7.1)	0.43
PLT, 10^9 ^/L (median, IQR)	346 (294–388.8)	326 (265.8–391.5)	0.41	352 (256–391)	272 (224–358)	0.04

IQR, interquartile range; CRP, C-reactive protein; FC, fecal calprotectin; WBC, white blood cell; ESR, erythrocyte sedimentation rate; PLT, platelet.

### Growth

The growth parameters are summarised in Table 4. Height Z-scores increased significantly from baseline to 12 months (−0.37, IQR: −0.7 to 0.36 vs. 0.22 IQR: −0.3 to 0.95, *p* = 0.0076). Weight Z-scores improved from baseline to 12 months (−0.82, IQR: −1.17 to 0.28 vs. −0.26 IQR: −0.65 to 0.26, *p* = 0.5849), the change was not significant. There was no significant change in BMI Z-score.

**Table 4:** Summary of growth parameters, expressed as Z-scores, at baseline and at 12 months.

	Baseline	12 months	*P* value
Height (median, IQR)	−0.37 (−0.7 to 0.36)	0.22 (−0.35 to 0.95)	0.0076
Weight (median, IQR)	−0.82 (−1.17 to 0.28)	−0.26 (−0.65 to 0.26)	0.5849
BMI (median, IQR)	−1.34 (−2.34 to 0.08)	−1.4 (−1.74 to 0.03)	0.9405

IQR, interquartile range; BMI, body mass index.

### Perianal disease

Five patients had perianal disease at diagnosis and four had perianal disease activity before treatment with UST. One patient experienced an exacerbation of perianal disease and no improvement in diarrhea after treatment with UST and one VEO-IBD patient with severe perianal disease also did not improve satisfactorily with the use of UST. However, perianal disease in the other two patients improved significantly, and perianal disease did not occur in the other patients.

### Stricturing disease

Eight patients had significant intestinal stenosis at the time of initial UST treatment, and four of them had colorectal stenosis. After UST treatment, one case of colon stenosis improved from multiple stenosis to one stenosis, while the other three cases had no significant improvement. Among the four children with small intestine stenosis, three of them underwent capsule endoscopy stuck in the small intestine. All of the four children with intestinal stenosis improved to varying degrees after UST treatment, and two of the children had their capsules excreted in the stool after about three months of UST treatment.

### Drug safety

Adverse events that were potentially related to therapy were reported in eight (50%) children, including one clostridium difficile infection in one patient (treated with oral antibiotics), one otitis media in one patient, and nine events of upper respiratory tract infection in eight patients. All adverse events were mild and no serious adverse events were noted with UST. During maintenance, adverse events were not the cause of medication cessation. In contrast, two patients discontinued UST due to the poor clinical response.

### Drug concentrations and antidrug antibodies

Of the 16 patients included in this study, 11 (69%) had UST drug concentrations and antidrug antibody levels tested (HeRui IBD, Suzhou, China) at a median time of 26 (24–43) weeks after UST initiation. Of the 11 patients, none had antibodies detected and concentrations ranged from 0.9 to 20 ug/ml. Nine patients had trough concentrations at weeks 24–32—median trough concentration for the patients was 10.3 (5.35–14.3) ug/ml. The median trough concentration for those in clinical remission was 13.65 (8.15–18.55) ug/ml compared with 5.5 (3.55–12.35) ug/ml for the patients not in clinical remission at weeks 24∼32 (*p* = 0.128). Of the remaining two patients, one had a trough concentration of 11.5 ug/ml at week 82 and one had a trough concentration of 0.9 ug/ml at week 52.

## Discussion

Compared with adults, pediatric patients with CD have fewer drug choices, rapid disease progression, more difficult treatment, higher cost, poor prognosis, and need more therapeutic drugs to choose. Despite the increasing number of treatments available, anti- TNF-α drugs remain the only proven and approved biologic therapy for pediatric patients with moderate to severe CD (2, 3). However, the treatment of CD with anti-TNF-α may lead to drug allergy, no response or secondary loss of response (4). Therefore, anti-TNF-α agents cannot meet the therapeutic needs of all pediatric patients with CD. The efficacy of UST for adult CD patients has been reported in the IM-UNITI study. 46.9% of patients achieved steroid-free remission and 10.9% achieved an endoscopic remission at week 44 (10, 17).

The regimen of UST for pediatric CD patients is not explicit. The most common regimen is an induction doses with 6 mg/kg intravenously and maintenance with 90 mg every 8–12 weeks, subcutaneously. Sandborn et al. reported that the high dose of UST therapy can improve the clinical response rate and clinical remission rate of CD patients (18). A multicenter retrospective study reported that 66% of patients recaptured response following treatment intensification with UST 90 mg every 4 weeks (19). Dayan et al. reported the rates of steroid-free remission at week 52 (60%) (20). Their regimen consisted of an intravenous induction dose followed by subcutaneous maintenance doses every 8 weeks; meanwhile, interval shortening to every 4 weeks or re-induction of UST was required in 39% of patients. In addition, the study also showed that bio-naïve patients were significantly more likely to achieve steroid-free remission than bio-exposed patients (20), suggesting that more aggressive UST therapy is needed in bio-exposed patients. Based on the above results, this study adopted a more aggressive regimen of UST. All patients were decision to prescribe UST was at the discretion of the prescribing physician.

Currently, data on the efficacy and safety of UST in the pediatric population are limited, and results vary widely among studies. This study reports on real-life experience using UST for the treatment of CD in a pediatric and young-adult patients from multiple IBD referral centers. Our data demonstrate that the steroid-free clinical remission rates were 41.7% at weeks 24–32, 75% at weeks 48–56 and clinical response rate were 66.7% at weeks 24–32, 87.5% at weeks 48–56. During the follow-up period, the endoscopic response rate and endoscopic remission rate were 45.5% and 18.2%, respectively. This suggests that pediatric and young adult patients could benefit from UST as either a primary or secondary biologic therapy for the induction, or maintenance of remission of CD. However, there were only few reports regarding the use of UST for pediatric patients with CD. A retrospective multicenter cohort study for pediatric patients reported that twelve of 44 (27.3%) patients achieved steroid-free remission at 12 months. In their study, induction therapy was given subcutaneously (21). Another single-center retrospective study of children from Japan reported the steroid-free clinical remission rates were 59% at week 26, 50% at week 52 (22). Patients in their study received an induction dose of 6–9 mg/kg intravenously, followed by subcutaneous injections every 8–12 weeks. The higher clinical response and remission rates in our study may be related to our more aggressive regimen of UST. In our study, all patients received a first induction dose intravenously, and then according to the disease activity and the wishes of their families, five patients followed by a subcutaneous infusion for maintenance every 8–12 weeks. Other eleven patients received an intravenous infusion for maintenance. So, we divided the results by different regiments between maintenance subcutaneously group and maintenance intravenously group to determine the effect of different maintenance methods on the treatment efficacy. The results showed there were no significant differences in clinical and biological indicators between the two groups (Supplementary Table S1), which may be limited by the small sample size. In addition, 68.75% of our cohort failed previous biologic treatments. Bio-naïve patients have a clinical remission rate of up to 66.7% at weeks 24–32. However, only a minority of our cohort were bio-naïve, so more date on exposure are needed to confirm this finding.

Traditional serological inflammatory markers such as CRP, ESR, albumin, and hemoglobin are associated with the disease activity of CD (23), Mallory et al. showed that CRP, ESR and albumin in most children with CD were significantly improved after UST treatment (21). In our study, CRP and ESR decreased and albumin and hemoglobin increased in most patients compared with baseline, which is consistent with the results of other studies. Many studies have shown that fecal calprotectin elevation is highly correlated with endoscopic disease activity, which can be used to evaluate the therapeutic effect of CD (24, 25). In this study, fecal calprotectin was significantly decreased in all patients, which also indicated the effectiveness of UST for the children with CD.

Compared with adult patients, children with IBD have a higher proportion of malnutrition which affects children’s growth and development (26). Mallory et al. showed that albumin and prealbumin of children were significantly improved after UST treatment, and their body weight increased significantly (21). In our study, the height Z-scores and weight Z-scores of patients increased compared with the baseline. Although there was no significant difference in the weight Z-scores, which may be limited by the small sample size.

Perianal disease seriously affect patients’ quality of life. Adult studies have shown that in adult CD patients treated with UST, the anal fistula remission rate over 24 weeks was 37.5% (27). In our study, the remission rate of anal fistula at 48–56 weeks was 2/4 (50%), which was higher than that in other studies, which may be related to the small sample size, and it is necessary to further expand the sample size to observe the effect of UST treatment of CD on perianal disease in children. UST is a fully humanized IgG1 monoclonal antibody that binds with specificity to the p40 protein subunit of interleukins IL-12 and IL-23 blocks the pathway mediated by downstream Th1 cells and Th17 cells, which reducing TGF-β and IL-17/22 production, thereby affecting myofibroblast formation and slowing down intestinal fibrosis (10, 11, 28). Our study showed that 5/8 (62.5%) of the children had varying degrees of improvement in intestinal stenosis, indicating that patients with stenosis can benefit from UST treatment.

Therapeutic drug monitoring (TDM) with UST is at its infancy. The clinical utility of TDM with non-anti-TNF mechanisms of action is not clear. A review of novel biologics for TDM in IBD recommended that UST concentrations of 3–7 ug/ml at week 8 and 1–3 ug/ml during maintenance have been associated with improved outcomes and dose optimization generally improves clinical outcomes in those with partial response or loss of response (29). In a recent *post hoc* analysis of UNITI studies (UNITI 1,2), Adedokun et al. reported that serum concentrations of UST during induction were correlated to the dose administration and therapeutic effect (30). The median trough concentration at weeks 24–32 in our study was 10.3 (5.35–14.3) ug/ml, and the median trough concentration for those in steroid-free remission was much higher than that patients who not in steroid-free remission at weeks 24–32. Although there was no statistical difference, this may be related to the small sample size. In addition, none of our patients had detectable anti-drug antibodies.

The safety of biologics in the treatment of CD in children is the most concerned issue for pediatricians and their families. A meta-analysis showed that 27% patients experienced adverse events, and serious adverse events was about 8.9%. Among the serious adverse events, infection was the most common, the incidence of tumor was 0.29%, and no tumor was reported in children (31). In this study, the patients were treated with a more active UST regimen. Although eight patients (50%) reported potentially treatment-related adverse events, all of the adverse events were mild and no children were discontinued as a result. However, the sample size is small, and it is necessary to further expand the sample size and prolong the observation time to obtain more reliable safety data of UST treatment of CD in children.

To date, UST treatment in children with monogenic IBD has been limited (32, 33). Of our two monogenic IBD patients, the one with BTK gene deficiency achieved a clinical response at 24 weeks, while the child with IL-10 gene deficiency did not achieve a clinical response at 24 weeks. Children with monogenic IBD face more difficult treatment challenges than those with non-monogenic. Biologics may be a viable option for partial monogenic IBD, but this needs to be validated with more research. Our study expands the experience of UST in the treatment of monogenic IBD.

In conclusion, our experience with the use of UST suggest that UST is efficacious and safe in pediatric CD patients. Given the real experience reported in our study and the various indications of use and phenotypes, it should be considered a viable treatment option for pediatric CD patients.

## Data Availability

The original contributions presented in the study are included in the article/**Supplementary Material**, further inquiries can be directed to the corresponding author.
