# Supplementary material for: Ustekinumab in pediatric patients with Crohn’s disease: safety, and efficacy results from a multicenter retrospective study in China
Source: Front Pediatr. 2024 Apr 11;12:1371322. doi: 10.3389/fped.2024.1371322 (PMC11043477; doi:10.3389/fped.2024.1371322)
Supplement: Supplementary file 1 [file Table1.pdf]

Supplementary Table S1 Summary of clinical indicators at baseline , at 24~32 weeks and at 48~56 weeks between SC with IV maintenance therapy

	Baseline			24~32weeks			48~56weeks		
	SC	IV	P Value	SC	IV	P Value	SC	IV	P Value
wPCDAI (median, IQR)	42.5(12.5-48.8)	42.5(7.5-50)	0.97	10(5-21.3)	15(0.5-25)	0.85	10(1.3-60.8)	6.3(1.3-9.4)	0.80
CRP, mg/L (median, IQR)	10(1.3- 60.8)	6.6(2.3-39.4)	0.71	1(0.5-8)	3.1(0.5-21.2)	0.42	4.1(0.5-9.5)	2.3(0.5-8)	0.88
ESR, mm/h (median, IQR)	27(19.5-41.5)	62(32-87)	0.052	12(8-20)	38(8-64)	0.15	13.5(5-19.8)	29(5-72)	0.41
FC, ug/g (median, IQR)	342(154.6-564.8)	398.9(262.5-824.1)	0.44	74(44-230)	265.7(49.68-447)	0.22	55.1(37.9-145)	64.9(41.7-189.3)	0.53
Albumin, g/L (median, IQR)	42(37.3-43.9)	37.5(35.8-41)	0.27	41(38.4-43.2)	40.1(37.9-42.5)	0.74	43.5(38.1-45.3)	44.3(42.6-45)	0.99
Hemoglobin, g/L (median, IQR)	137(105.5-147)	112(102-141)	0.42	140(115-142.5)	129(109-138)	0.28	146(133-158.3)	124(120-134)	0.21
WBC,10^9/L (median, IQR)	6.9(4.7-9.5)	6.6(4.7-10.7)	0.99	6.3(4.8-8.5)	8.3(5.7-8.7)	0.38	5.8(4.6-9.8)	6.3(5.9-7.2)	0.79
PLT, 10^9/L (median, IQR)	306(243-414.5)	349(314-391)	0.38	265(244-346.5)	349(295-467)	0.15	240(205.3-321.3)	325(258-391)	0.16

IQR, interquartile range; IV, intravenous; SC, Subcutaneous; wPCDAI, weighted Pediatric Crohn’s Disease Activity Index; CRP,C-reactive protein; FC, fecal calprotectin; WBC, white blood cell; ESR, erythrocyte sedimentation rate; PLT, platelet.
